# P-841. Impact of a Pediatric Reflex Urine Testing Algorithm Across a Multihospital Network

**DOI:** 10.1093/ofid/ofaf695.1049

**Published:** 2026-01-11

**Authors:** Katarzyna M Stoj, David Kuang, Barbara Ross, Adam L Gouveia, Xiaoyue Ma, Lisa Saiman, Harjot K Singh, Jennifer Lee, Karen P Acker

**Affiliations:** Columbia University Irving Medical Center and Weill Cornell Medicine NewYork Presbyterian, Hoboken, NJ; NEW YORK PRESBYTERIAN, HOBOKEN, New Jersey; NewYork-Presbyterian Hospital, New York, NY; NewYork-Presbyterian, Brooklyn, NY; Weill Cornell Medicine, New York, New York; Columbia University Irving Medical Center, New York, NY; Weill Cornell Medicine, New York, New York; Columbia University Irving Medical Center, New York, NY; Weill Cornell Medicine/New-York Presbyterian, New York, NY

## Abstract

**Background:**

Inappropriate urine cultures (UCx) performed in children lead to false-positive results, asymptomatic bacteriuria detection, unnecessary antibiotic use, and increased healthcare costs. To reduce unnecessary UCx in our hospital network, we implemented a diagnostic stewardship initiative utilizing reflex urine testing in July 2023 for children > 24 months.

**Figure 1.**
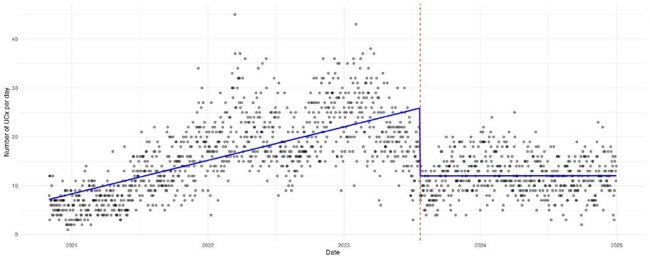
Urine Cultures Performed Per Day Over Time An ITS analysis was performed of daily UCx counts to measure the impact of reflex testing on the number of UCx sent.

**Table 1.**
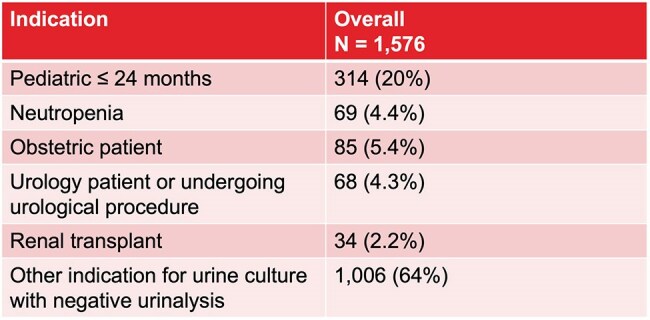
Indications for Opting Out of Reflex Urine Testing

The following indications were selected by providers for opting out of the reflex urine testing algorithm.

**Methods:**

A retrospective cohort study was conducted in children > 24 months to 18 years who had urinalyses (UAs) and UCx sent across inpatient and ED settings in 8 NYP hospitals before (11/2020–7/2023) and after (7/2023–12/2024) implementation of reflex urine testing. Per the reflex algorithm, UCx are performed only if the UA has ≥ 10 WBC/hpf and the patient has no high-risk conditions (age ≤ 24 months, pregnancy, neutropenia, urological procedures, and renal transplant). Median number of monthly UCx pre- and post-implementation were compared using Wilcoxon rank-sum test. Interrupted time series (ITS) analysis assessed the UCx trends between periods. UCx positivity rates, number of UCx processed based on UA results, and reasons for opting out of reflex testing were evaluated.

**Results:**

Over 32 months pre-implementation, 51,391 UAs and 16,433 UCx were sent, and in the post-implementation period of 17 months, 30,258 UAs and 6,339 UCx were performed. Median number of monthly UCx decreased from 530 (IQR 330, 631) to 369 (IQR 332, 393) (p < 0.010), a 30% reduction. ITS analysis demonstrated a sustained post-implementation reduction of UCx per day (-0.01, p < 0.001) (Figure 1). UCx positivity increased from 6% pre- to 9% post-implementation (p < 0.001). Following implementation, 11,367 UAs were processed under reflex testing; 9791 (86.1%) opted in, and 1576 (13.9%) opted out for reasons listed in Table 1. Of the 9,791 UAs that opted in for reflex testing, 8,360 (85.3%) had < 10 WBC, with 26 (0.3%) having UCx done per providers’ request. 1,383 (14.1%) had ≥ 10 WBC, with 73 (5.3%) not undergoing UCx due to specimen handling errors.

**Conclusion:**

Pediatric reflex urine testing led to a significant reduction in UCx ordering while increasing culture positivity. Despite minor errors, opportunities remain to further limit unnecessary UCx by addressing opt-outs for reflex urine testing through education and enhanced decision support.

**Disclosures:**

Lisa Saiman, MD MPH, AstraZeneca: Grant/Research Support|Merck: Grant/Research Support

